# O057: Clinical evaluation of the antiseptic efficacy and local tolerability of a polihexanide-based antiseptic in comparison to a chlorhexidine-based antiseptic on intact skin

**DOI:** 10.1186/2047-2994-2-S1-O57

**Published:** 2013-06-20

**Authors:** FHH Brill, D Egli-Gany, M Hintzpeter

**Affiliations:** 1 Dr. Brill + Partner GmbH Instiute for Hygiene and Microbiology, Hamburg, Germany; 2 Private Researcher, Horgen, Switzerland; 3 B. Braun Melsungen AG, Melsungen, Germany

## Introduction

The antiseptic agent chlorhexidine is internationally widely-used and well-accepted for skin and wound antiseptics. In recent years, the agent polihexanide is gaining importance for similar purposes. Both agents are biguanides and therefore similar characteristics may be expected.

## Objectives

The primary objective of this study was to compare the antimicrobial efficacy of polihexanide 0.02 % and 0.04 % with chlorhexidine 0.05 % after 30 min of treatment of healthy skin. The secondary objectives were to evaluate the local tolerability and the antimicrobial efficacy after 5 and 10 min contact time.

## Methods

The study was performed as a double-blind, randomized, comparator-controlled, 3-arm, crossover study on 20 healthy volunteers with intact skin in a phase 1 study unit.

Test areas of 5 cm^2^ on the subjects‘ arms were treated with investigational and reference products using a polyurethane swab. Skin swabs were taken before and after treatment for quantitative microbial evaluation.

The main outcome measure was the log_10_ reduction factor (RF) of colony-forming units (cfu) on the skin after 30 minutes of treatment. Further endpoints were the RF after 5 and 10 minutes and the local tolerability.

## Results

No statistically significant difference was seen between the test products polihexanide 0.02 %, 0.04 % and the comparator, chlorhexidine 0.05 % after 30 min of treatment (p > 0.1). The analysis of the exposure times of 5 and 10 minutes revealed that the antiseptic efficacy of polihexanide 0.02 % is statistically significantly lower than that for the comparator chlorhexidine; polihexanide 0.04 % on the contrary not. No statistically significant differences in local tolerability were observed between the three products [1].

## Conclusion

The results of this clinical study indicate that polihexanide is a suitable alternative to chlorhexidine and shows a comparative efficacy on the skin.

## Disclosure of interest

None declared.
